# A Delphi consensus on the management of Spanish patients with osteoporosis at high risk of fracture: OSARIDELPHI study

**DOI:** 10.1007/s11657-023-01318-7

**Published:** 2023-08-23

**Authors:** Luis Arboleya, Jose Manuel Cancio-Trujillo, Celia Chaves, Enric Duaso-Magaña, Manuel Mesa-Ramos, Jose Manuel Olmos

**Affiliations:** 1 Rheumatology Service, Hospital Universitario Central de Asturias, Oviedo, Asturias Spain; 2 Departament of Geriatric Medicine and Palliative Care, Badalona Serveis Assistencials, Catalonia, Spain; 3 Medical Affairs Department, STADA, Barcelona, Spain; 4 Acute Geriatric Unit, Geriatric Service, Hospital de Igualada, Barcelona, Spain; 5 Orthopedic Service. Hospital Valle de los Pedroches, Pozoblanco, Córdoba, Spain; 6 Internal Medicine Service, Hospital Universitario Marqués de Valdecilla-IDIVAL, Universidad de Cantabria, Santander, Cantabria Spain

**Keywords:** Consensus, Delphi method, Osteoporosis, High-risk fracture, Treatment, Fracture-liaison service (FLS) unit

## Abstract

**Summary:**

The OSARIDELPHI study evaluated the level of agreement between specialists in osteoporosis regarding the management of patients with high-risk fractures in Spain. The results provide expert-based recommendations for prevention, diagnosis, and treatment related to fracture risk. Therefore, the study facilitates clinical decision-making for managing this patient’s profile.

**Purpose:**

To evaluate the level of agreement between specialists in osteoporosis regarding the management of patients with high-risk fractures in Spain.

**Methods:**

A two-round Delphi study was performed using an online survey. In round 1, panel members rated their level of agreement with assessments on a 9-point Likert scale. Item selection was based on acceptance by ≥ 66.6% of panel experts and the agreement of the scientific committee. In round 2, the same panelists evaluated non-consensus items in round 1.

**Results:**

A total of 80 panelists participated in round 1; of these, 78 completed the round 2 survey. In round 1, 122 items from 4 dimensions (definition of fracture risk: 11 items, prevention and diagnosis: 38 items, choice of treatment: 24 items, and treatment-associated quality of life: 49 items) were evaluated. The consensus was reached for 90 items (73.8%). Panelists agreed that categorizing high risk, very high risk, or imminent risk determines secondary prevention actions (97.5%). Experts agreed that treatment with bone-forming drugs should be considered in case of a very high risk of fracture, and a sequential change to antiresorptive drugs should be made after 1–2 years (97.5%). Panelists also recommended corrective action plans for non-adherent patients to improve adherence (97.5%). A total of 131 items were finally accepted after round 2.

**Conclusion:**

This Delphi study provides expert-based recommendations on clinical decision-making for managing patients with osteoporosis at high risk of fracture.

**Supplementary Information:**

The online version contains supplementary material available at 10.1007/s11657-023-01318-7.

## Introduction

Osteoporosis is a skeletal disease characterized by decreased bone mass and deterioration of bone microarchitecture, leading to increased fragility and consequent fracture risk [1]. It is the most common metabolic bone disease in Western countries, affecting 25–32% of Spanish women over 50 years and almost 50% over 75 years [2]. In the absence of more recent updates on disease prevalence data, in 2010, it was estimated that in Spain, there were around 1.9 million cases in women and 400,000 cases in men, with an estimated cost of 2.8% of total health expenses [3, 4]. The incidence has increased in the following years, as well as the direct costs of incident fractures. Thus, in 2019, it was calculated that in Spain, there were around 2.9 million cases, of which almost 80% were women, with an estimated cost of 3.8% of total healthcare expending [5, 6].

Fragility fractures are a significant cause of disability, morbidity, and mortality in the population [7]. Hence the importance of prevention and early diagnosis, especially in patients at high risk and very high risk of fracture. A previous fracture due to this pathology, especially recent in time, is considered one of the risk factors identified for fragility fractures [8–12]. However, patients perceive them to be due to the environment or accidental falls. They often do not consider that they should be screened, observe preventive strategies, or even receive therapy for osteoporosis [13]. Recently, different studies analyzed the risks of imminent fractures in various cohorts in Spain [14, 15]. These studies showed that higher risks occur in women aged ≥ 80 years, and the 10-year risk levels were estimated between 1.8 and 21.5% in women and between 0.7 and 10.8% in men using the QFRACTURE tool [14]. More recently, an estimate of the incidence ratio of subsequent fractures in the 3 years following an initial fracture observed that 3.2% of women patients ≥ 50 years with a previous fracture experienced a new fracture every following year [15]. Despite all these data, underdiagnosis and under-treatment of osteoporosis are quite common, as is the case of vertebral fractures, which are the most frequent [16–18]. Vertebral fractures are often asymptomatic or mildly painful, and radiographic detection routines are scarce [17].

Optimal management of this patients’ profile has also impacted the quality of life of people with osteoporosis. Due to the presence of comorbidities, they are often polymedicated, usually associated with lower adherence to treatment, in addition to the dosage of the drugs and the side effects of the different therapies [19]. Indeed, patient follow-up program strategies are critical for those at increased risk of fracture to improve long-term treatment persistence and prevent secondary fractures [20–22].

Consequently, it is relevant to establish consensus among specialists in managing patients with osteoporosis to have effective strategies for prevention and diagnosis. It is also important to adapt the best therapy to each patient profile and to optimize the different lines of treatment over time, from the early stages of the disease. It is essential to consider the current availability of therapies, particularly biological treatments, in patients at high risk of fracture. In this sense, updated guidelines and recommendations from a number of organizations, including European Society for Clinical and Economic Aspects of Osteoporosis (ESCEO) and the International Osteoporosis Foundation (IOF) are available [23–25]. However, these recommendations need to be adapted locally, taking into account the characteristics of the population and the specific health resources at regional level.

Based on this background, we conducted the OSARIDELPHI study (from the Spanish title: *Manejo clínico del paciente con OSteoporosis de Alto RIesgo de fractura en España: prevención y diagnóstico de la patología, opciones de tratamiento más adecuadas y su impacto en la calidad de vida del paciente*). This Delphi method study addressed multiple key questions and controversies related to managing the patient with osteoporosis of high-risk fracture. A two-Delphi survey was used to seek expert-based opinions to develop a set of consensus guidelines that may support physicians in clinical decision-making and improve real-world practice in this patient’s profile.

## Methods

### Study design

The OSARIDELPHI project was a nationwide Spanish multicenter 2-round Delphi study to seek expert opinion on the clinical management of the patient with osteoporosis of high risk of fractures in Spain.

The approval of the Institutional Review Board (IRB) or by the equivalent ethics committee(s) was not required as this Delphi study does not involve human subject research. No patient data were collected for this study, which was based entirely on the feedback and opinions provided by experts.

The Delphi process is a widely accepted scientific method of systematic information collecting from a group of experts (termed the Delphi expert panel) on controversial or complex topics [26]. Each panel expert provides opinions individually and anonymously without the biasing effect of dominant individuals or group pressure [27–29]. The Delphi process ends when an agreement has been reached on the discussed topics.

The Delphi project was carried out in six steps: (1) literature review (by the scientific committee); (2) discussion and questionnaire domain/item generation by the scientific committee in a teleconference meeting; (3) selection of the Delphi expert panel and the invitation to candidates to participate in Delphi process; (4) domain/item set evaluation by the panel experts through 2 rounds using an online platform through a web platform (two-round Delphi approach); (5) final discussion of items that did not reach consensus in preceding rounds among the scientific committee experts; (6) final consensus analysis.

### Delphi process

#### Selection of Delphi participants

The expert scientific committee comprised five specialists experienced in managing patients with osteoporosis of high-risk fracture and recognized experts in the field.

A total of 80 specialists from hospitals distributed across Spain were invited to participate in the project as members of the Delphi expert panel in both round 1 and round 2 of the Delphi process. The panel experts were selected based on their extensive experience and knowledge of the management of patients with osteoporosis.

The expert panel members were provided with an informative leaflet outlining the aims and the study procedure and including an electronic link to the online survey. Experts received personalized access to the online survey. Panel members participated in the project through 2 rounds of the Delphi process using the online questionnaire. The purpose of the expert panel was to reach a consensus based on the current clinical evidence and their daily practice in and knowledge of the management of patients with osteoporosis.

#### Selection of Delphi questionnaire dimensions and items

The scientific committee carried out a systematic literature review regarding managing patients with osteoporosis of high-risk fractures, focusing on current controversial and unmet topics. After a careful and critical review of the selected literature and based on their knowledge of the clinical management of the pathology, the scientific committee developed the first set of domains and items for the Delphi questionnaire in a teleconference meeting.

##### Round 1

The members of the Delphi expert panel were asked between July and September 2021 to rate their level of agreement with each questionnaire item on a 9-point Likert scale from 1 (completely disagree) to 9 (completely agree). Each item was categorized according to the scores as rejected (scores 1–3), undetermined (scores 4–6), or accepted (scores 7–9). Panelists were also encouraged to provide comments after scoring each item using open-text comment fields included in the online survey.

After analyzing the data obtained from the first Delphi round, the scientific committee experts participated in a teleconference meeting, where the Delphi survey results were presented and discussed. Item selection was based on the acceptance of questionnaire items by ≥ 66.6% of the expert panel and the agreement of the scientific committee. Statements not achieving 66.6% agreement were removed or modified according to the feedback provided by the expert panel. All statements were assessed, given the experts’ suggestions. After round 1 was completed and the expert comments had been summarized, amendments were made to some questionnaire items. Where necessary, new items were generated and included. The updated questionnaire was redistributed to the panelists for round 2.

##### Round 2

In round 2, the same panel members were asked between February and March 2022 to evaluate the list of items that did not meet consensus from round 1, using the same voting method described for the initial round. For this evaluation, the panel members were provided with a summary of the opinions issued anonymously by the participants in the first round, in addition to any other information that the scientific committee deemed appropriate to make available to the panelists to achieve consensus. Thus, the panelists could reflect upon the group’s responses after the first round and re-evaluate the non-consensus items in view of the other experts’ feedback.

After analysis of the responses as described for round 1, the statements not meeting expert agreement were retained for discussion.

##### Concluding round

The concluding round comprised a teleconference meeting among the scientific committee experts to assess those items that did not reach a consensus in round 2. The scientific committee members discussed the non-consensus items until an agreement was reached to retain or eliminate the item from the final consensus guidelines.

### Statistical analysis

A descriptive statistical analysis of the data obtained from the expert panel’s assessment of the Delphi questionnaire items in rounds 1 and 2 was conducted. The distribution of frequencies of panel responses on the 9-point scale was calculated to establish the level of consensus for each questionnaire item.

A descriptive statistical analysis of the characteristics of the Delphi expert panel was also performed, including calculation of central tendency and dispersion (mean ± standard deviation, median and interquartile range) for quantitative variables and frequencies and valid percentages for qualitative variables.

The statistical analysis was performed using the Statistical Package for the Social Sciences (SPSS) version 18.0 (SPSS Inc., Chicago, IL, USA).

## Results

### Panel experts

A total of 80 specialists from 69 centers distributed throughout Spain agreed to participate as Delphi panel experts. All 80 experts participated in round 1, whereas 78 completed the round 2 survey. The characteristics of the Delphi experts are summarized in Table 1. Briefly, most participants were associate physicians (*n* = 73, 91.3%) in public hospitals (*n* = 74; 92.5%) with a median (range) of 16.5 (9.0–24.5) years of professional experience. Most participants were rheumatologists (*n* = 46; 57.5%), followed by traumatologists (*n* = 18; 22.5%). Remarkably, 34 (42.5%) and 20 (25.0%) participants indicated that they had in their hospitals a multidisciplinary team and fracture-liaison service, respectively, as resources related to the management of osteoporosis patients at risk of fracture.

**Table 1:** Characteristics of the Delphi expert panel

Characteristics	(*N* = 80)
Age, median (range), years	44.5 (36.0–54.8)
Gender, male, *n *(%)	43 (53.8)
Professional experience, median (range), years	16.5 (9.0-24.5)
Hospital position, *n *(%)	
Attending physician	73 (91.3)
Service head	4 (5.0)
Other	3 (3.8)
Research activity, *n* (%)	
Investigator	31 (38.8)
Without research activity	30 (37.5)
Professor	11 (13.8)
Other	8 (10.0)
Specialty, *n* (%)	
Rheumatology	46 (57.5)
Traumatology/orthopedic surgery	18 (22.5)
Other	7 (8.75)
Rehabilitation	6 (7.5)
Internal Medicine	3 (3.75)
Type of hospital, *n* (%)	
Public hospital	74 (92.5)
Private hospital	5 (6.3)
Other	1 (1.3)
Resources related to the management of patients at risk of fracture, *n* (%)	
Multidisciplinary team	34 (42.5)
Fracture-liaison service (FLS)	20 (25.0)
Patient care program	19 (23.8)
Other	7 (8.8)

### Results from round 1 and round 2

**Fig. 1:**
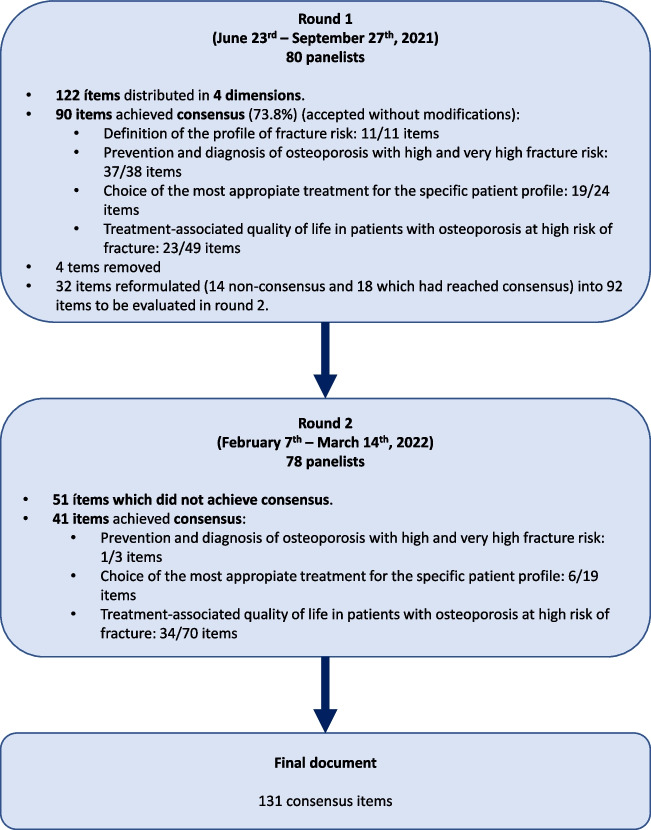
Results of the Delphi study

Figure 1 illustrates the results of the Delphi study. In round 1, panel members evaluated 122 items from the following dimensions:

1. Definition of the profile of fracture risk: 11 items
2. Prevention and diagnosis of osteoporosis with high and very high fracture risk: 38 items, subdivided into two sections (underdiagnosis and diagnosis/prevention)
3. Choice of the most appropriate treatment for the specific patient profile: 24 items, subdivided into four sections (availability of current therapeutic alternatives, treatment lines/optimization, biosimilar availability, and adverse events)
4. Treatment-associated quality of life in patients with osteoporosis at high risk of fracture: 49 items, subdivided into two sections (adherence to treatment and patient care programs)

The consensus was reached for 90 items (73.8%), which were accepted without modifications. After the scientific committee meeting, 4 items were removed, and 32 (14 non-consensus items and 18 items that had reached consensus) were reformulated and expanded to 92 items to be evaluated in round 2.

The items that did not meet consensus or were reformulated after round 1 were put forward for inclusion in round 2, along with accompanying comments. Forty-one items reached an agreement in round 2. Fifty-one items did not achieve consensus in round 2, and after discussion, the scientific committee experts decided to maintain non-consensus. At the end of the Delphi process, a total of 131 items were finally retained.

Supplementary Tables 1-4 summarize the results from the Delphi process and the level of agreement after the 2 rounds for the statements related to the definition of the fracture risk (Supplementary Table 1), the prevention and diagnosis of osteoporosis patients with high and very high–risk fracture risk (Supplementary Table 2), the choice of the appropriate therapy for the specific patient profile (Supplementary Table 3), and the treatment-associated quality of life in patients with a high risk of fracture (Supplementary Table 4).

To simplify the interpretation of the results, a set of recommendations was developed with the most relevant items that reached expert consensus in the Delphi process (Table 2).

**Table 2:** Expert-based Delphi consensus recommendations for the management of patients with osteoporosis at high risk of fracture

Definition of profile of fracture risk	1. The categorization of the risk of fracture due to osteoporosis is determined by the history of previous osteoporotic fractures, a recent major fragility fracture, the family history of hip fracture, the treatment with high-dose glucocorticoids, the ten-year fracture risk probability, and the data of the bone densitometry
	2. The categorization of the osteoporotic fracture risk as high risk, very high risk or imminent risk determines the implementation of secondary prevention actions
Prevention and diagnosis of osteoporosis with high and very high fracture risk	3. More effort is needed in the diagnosis of osteoporosis at high-risk fracture, since a high number of patients who have suffered a first symptomatic fragility or vertebral fragility fractures do not undergo secondary prevention.
	4. Assessing and follow-up of patients with osteoporosis at high and very high risk of fracture should be done systematically and proactively to manage the disease more efficiently.
	5. Clinical assessment for early diagnosis should include information regarding personal history of fragility fractures, parental history of hip fractures, history of treatment with glucocorticoids at high doses for more than 3 months, secondary causes of osteoporosis, and history of falls in the past 3 months.
	6. To prevent new fragility fractures, recommendations, and changes in lifestyle habits, antiresorptive or bone-forming therapies, calcium and vitamin D supplementation and rehabilitation and long-term patient follow-up program should be carried out.
Choice of the most appropriate treatment for the specific patient profile	7. In the case of the category of very high risk of fracture, treatment with bone-forming drugs should be considered, and a sequential change to antiresorptive drugs should be assessed after one or two years.
	8. The monitoring criteria for the different therapeutic alternatives available are defined by the improvement in bone densitometry and bone remodeling markers, the absence of new fractures, and the decrease in the risk of new fractures.
Treatment-associated quality of life	9. The team of fracture-liaison service (FLS) unit should consist of traumatologists, rheumatologists, specialists in geriatrics, specialist nurses, primary care physicians, physiotherapists, rehabilitators, nutritionists, and specific patient support staff.
	10. The FLS units should monitor the management of patients with osteoporosis at high and very high risk of fracture at the level of hospitals, primary care centers and intermediate care centers (if available).
	11. The FLS units should be coordinated by physician and nursing staff specialized in the management of patients with osteoporosis, primary care physicians, and intermediate care staff (if available).
	12. The primary care physicians for each patient with osteoporosis at high-risk fracture should be informed of the specific plan for the management of the patient.
	13. The training of the patient to handle treatment delivery devices should be carried out by nurses and specialized staff of a patient support program.

## Discussion

The results of this Delphi study demonstrate a high degree of consensus between experts involved in the management of osteoporosis patients with a high and very high–risk of fracture. This expert panel study provides insights on important topics such as the factors associated with more probability of high and very high–risk of fracture, the diagnosis, and the treatment choice for the specific patient profile.

Overall, according to the Delphi survey, experts agreed on the categorization of the fracture risk due to osteoporosis is determined by the history of previous osteoporotic fractures, a recent major fragility fracture, the family history of hip fracture, the treatment with high-dose glucocorticoids, the 10-year fracture risk probability, and the data of the bone densitometry. The IOF and ESCEO recommended that the risk of fracture should be expressed as absolute risk, i.e., the probability of fracture over a 10-year interval [30]. The assessment strategy to categorize risk was recently improved, including low and high risk and very high risk [23, 24]. Very high risk was defined as a fracture probability above the upper assessment threshold after a FRAX® assessment, including bone mineral density (BMD) if available [23, 24]. The panel of experts of the present Delphi survey also agreed that the implementation of secondary prevention actions is a consequence of this categorization. This result is in line with previous recommendations suggesting that preventive treatment given as soon as possible after fracturing in patients with very high fracture risk would avoid a higher number of new fractures and reduce the attendant morbidity [23, 24].

The panelists recommended follow-up systematically and proactively to the patients at high and very high risk of fracture (agreed in round 1 by 100% of the experts). In addition, experts, besides age and female gender, recommended including in the assessment the following risk factors: personal and parental history of fragility fractures (97.5%); history of treatment with glucocorticoids at doses ≥ 5 mg/day for ≥ 3 months (98.8%); causes of secondary osteoporosis (98.8%); history of falls in the past 3 months (92.5%), radiological imaging tests (92.5%); and BMD (97.5%). Another aspect related to risk assessment and categorization was recently highlighted as a new pivotal point in osteoporosis: FRAX® arithmetically integrating with novel risk factors [25, 31]. Accordingly, it is widely recognized that BMD alone for fracture risk assessment is less sensitive than algorithms, such as FRAX®, which incorporate risk indicators in addition to BMD [31].

The experts consistently agreed that treatment with bone-forming drugs should be considered in the very high fracture risk category. A sequential change to antiresorptive medications should be assessed after 1 or 2 years (97.5%). In addition, panelists recommended that the monitoring criteria for the different available alternatives of treatment should be defined by the improvement in bone densitometry (87.5%) and bone remodeling markers (78.8%), the absence of new fractures (93.8%), and the decrease in the risk of further fractures (95%). Regarding long-term treatment, panelists agreed that the duration of treatment with oral bisphosphonates, intravenous bisphosphonates, teriparatide, and denosumab is currently well defined at 5 (71.8%), 3 (69.2%), 2 (94.9%), and 10 years (71.8%).

Although osteoporosis is a well-recognized problem with a choice of widely available treatments, a large treatment gap exists [32]. Some treatments for osteoporosis (i.e., oral bisphosphonates, menopausal hormonal therapy, and selective estrogen receptor modulators) have suboptimal efficacy due to the difficulties in meeting treatment goals with such therapies in the highest fracture-risk patients [33]. The treatment stratification according to baseline fracture risk permits targeting the most effective treatments for this patient profile [34]. Specifically, guidance thresholds leading to distinguish high and very high fracture risk have optimized the use of anabolic agents [32, 34]. In patients at the highest fracture risk, treatment initiation with an anabolic (bone-forming) agent, such as teriparatide, followed by an antiresorptive to maintain the gains in bone mineral density, appears now a highly appropriate strategy to achieve a rapid and sustained reduction in fracture risk [8, 30]. In this sense, the availability of romosozumab and abaloparatide would be after the completion of the present Delphi study (conducted between July 2021 and March 2022). This recommendation has strong evidential support from recently published studies comparing anabolic vs. antiresorptive therapies [35–38]. These studies demonstrated a more rapid and significant fracture risk reduction with the former, compared with oral antiresorptive treatments alone [35–38]. However, these benefits must be maintained by following the anabolic with an antiresorptive drug [36, 39, 40]. Indeed, the evidence suggests that the treatment sequence is important, such that an anabolic agent given before an antiresorptive agent is more effective than the opposite sequence [41, 42]. This model suggests the need for physicians to be able to identify the patients who would most benefit from anabolic therapy [32].

The decision on when to stop antiresorptive treatment in a patient who has received a prior anabolic agent is complex. Recent data showed that discontinuation of up to a year might be acceptable in the case of previously alendronate- and zoledronate-treated patients [43]. In contrast, in the other agents (risedronate, ibandronate, raloxifene, teriparatide, denosumab, and romosozumab), the bone loss at the femoral neck and total hip is higher, indicating the importance of continuation of these antiresorptive agents [43]. Considering all these data, the consensus is that prolonged antiresorptive therapy will be necessary after anabolic treatment if the patient remains at high or very high fracture risk. However, if a patient is no longer at high or very high fracture risk, it may be possible to stop treatment for a maximum of 2 years, except for denosumab, due to the risk of rebound vertebral fractures [43].

This Delphi study also highlighted that experts agreed on the importance of the existence of fracture-liaison service (FLS) units in managing patients with high and very high fracture risk. According to experts, these FLS units should be composed of professionals who cover the different aspects crucial in managing this patient profile and with varying levels of specialization. However, there are differences in consensus regarding the current use of FLS (71.8% of the panelists stated that FLS units currently manage this patient profile) and what should happen (97.4% of the experts agreed that an FLS unit should monitor these patients). Notably, implementing FLS has increasing evidence since these units can improve access to better management and treatment to reduce future fractures [44–46]. A recent systematic review and meta-analysis evaluated the clinical impact of FLS implementation based on the results of several studies encompassing 48,045 patients [46]. The results suggested that FLS significantly improves the rates of dual-energy X-ray absorptiometry (DXA) scanning and antiresorptive therapy prescription and reduces new fracture rates [46]. Regarding possible controversies, a lack of consensus was observed on the current clinical application of bone remodeling markers in the early diagnosis of the disease (20.5%) and the prediction of fracture risk and bone mass loss (42.3%). The lack of consensus was also reflected in the simultaneous combination of a bone-forming drug with an antiresorptive treatment (50%). These results at the time of the Delphi survey reflect the absence of solid recommendations regarding using bone remodeling markers as a current diagnostic tool and of scientific evidence concerning the simultaneous combination of available therapies.

One of the limitations of the present study was a greater representation of specialists in rheumatology or traumatology among the participating experts compared to the rest of the specialties responsible for the management of patients with osteoporosis. Probably, the fact that there was no participation of primary care physicians explains the high percentage of experts in whose centers there were multidisciplinary units and FLS. In addition, it should be taken into account that the Delphi methodology itself means that the fact that experts express a high degree of agreement does not directly imply that a recommendation is necessarily effective. The results of the study represent the starting point for the development of recommendation documents and management guidelines. In summary, the results of this Delphi study suggested that, although the general lines of recommendations and suggestions are in line with the management recommendations established by, among others, the IOF or the ESCEO, an adaptation to the specific characteristics of the Spanish population and available health resources is needed. Specifically, regional differences can be observed in the implementation of FLS units, which may determine an adaptation of the recommendations to the Spanish reality.

## Conclusion

The expert consensus recommendations derived from this Delphi panel study may provide support and guidance on clinical decision-making regarding osteoporosis management in specific clinical patients’ profiles, specifically in real-world patients with high or very high fracture risk. Additionally, this consensus analysis may encourage discussion on controversial issues addressed in the consensus statements.

### Supplementary information

ESM 1

## Data Availability

The datasets used and/or analyzed during the current study are available from the corresponding author on reasonable request.
