# Supplementary material for: A Delphi consensus on the management of Spanish patients with osteoporosis at high risk of fracture: OSARIDELPHI study
Source: Arch Osteoporos. 2023 Aug 23;18(1):110. doi: 10.1007/s11657-023-01318-7 (PMC10447260; doi:10.1007/s11657-023-01318-7)
Supplement: Supplementary file 1 — ESM 1 [file 11657_2023_1318_MOESM1_ESM.pdf]

Supplementary Table 1: Results of the two-step Delphi process for the items regarding to the definition of the profile of fracture risk

Items	Accepted (scores 7-9) n (%)	Result of Delphi process
1 - 6. The categorisation of the risk of fracture due to osteoporosis is determined by:		
History of previous osteoporotic fractures	78 (97.5)	Agreed in round 1
Recent major fragility fracture	75 (93.8)	Agreed in round 1
Family history of hip fracture	69 (86.3)	Agreed in round 1
Treatment with high-dose glucocorticoids	78 (97.5)	Agreed in round 1
Ten-year fracture risk probability	70 (87.5)	Agreed in round 1
Bone densitometry	68 (85.0)	Agreed in round 1
7. The categorisation of the osteoporotic fracture risk is defined as low, high, and very high risk	75 (93.8)	Agreed in round 1
8. The categorisation of the osteoporotic fracture risk as high risk, very high risk or imminent risk determines the implementation of secondary prevention actions	78 (97.5)	Agreed in round 1
9. Sentinel fracture is an opportunity to categorise fracture risk during the secondary prevention.	76 (95.0)	Agreed in round 1
10. The probability of imminent fracture risk is defined as very high when a fracture triggers a cascade of new fragility fractures.	77 (96.3)	Agreed in round 1
11. Imminent fracture risk increases in relation to the increased number and severity of prevalent vertebral fractures	80 (100.0)	Agreed in round 1

Supplementary Table 2: Results of the two-step Delphi process for the items regarding to prevention and diagnosis of osteoporosis with high and very high fracture risk

Items	Accepted (scores 7-9) n (%)	Result of Delphi process
**Underdiagnosis**		
1. Osteoporosis is an underdiagnosed disease before a first fragility fracture occurs	77 (96.3)	Agreed in round 1
2. A high number of patients who have suffered a first symptomatic fragility fracture do not undergo secondary prevention	74 (92.5)	Agreed in round 1
3. A high percentage of vertebral fragility fractures are asymptomatic or undiagnosed	76 (95.0)	Agreed in round 1
**Diagnosis / Prevention**		
1. Assessing the risk of fragility fracture before the first fracture occurs is important to manage the disease more efficiently	79 (98.8)	Agreed in round 1
2. Follow-up of patients at high and very high risk of fracture should be done systematically and proactively	80 (100.0)	Agreed in round 1
3. Screening for early diagnosis should be performed regularly	76 (95.0)	Agreed in round 1
4-7. Clinical assessment for early diagnosis should include information on the following risk factors:		
Personal history of fragility fractures and parental history of hip fractures	78 (97.5)	Agreed in round 1
History of treatment with glucocorticoids at doses greater than 5mg/day for more than 3 months	79 (98.8)	Agreed in round 1
Causes of secondary osteoporosis	79 (98.8)	Agreed in round 1
History of falls in the past 3 months	74 (92.5)	Agreed in round 1
8-10. Clinical evaluation for early diagnosis should include the following diagnostic tests:		
Radiological imaging tests	74 (92.5)	Agreed in round 1
Bone densitometry	78 (97.5)	Agreed in round 1
Other laboratory tests	68 (85.0)	Agreed in round 1
11-13. Bone remodelling markers in osteoporosis currently have clinical application in:		
In the early diagnosis of the disease	16 (20.5)	Not agreed in round 2 after being reformulated(1)
The evaluation of therapeutic response	53 (67.9)	Agreed in round 2 after being reformulated(1)
Prediction of fracture risk and bone mass loss, and its correlation with bone densitometry	33 (42.3)	Not agreed in round 2 after being reformulated(1)
**Risk assessment**		
1. The risk of suffering a new fragility fracture increases in the 24 months following a previous fracture	78 (97.5)	Agreed in round 1
2-4. Specific protocols for the assessment and prevention of the risk of a new fracture should be followed in:		

Hip fractures	79 (98.8)	Agreed in round 1
Vertebral fracture	78 (97.5)	Agreed in round 1
Non-vertebral fractures (proximal humerus, distal forearm, tibia, pelvis, sacrum, ribs, among others)	75 (93.8)	Agreed in round 1
5-14. Fracture risk assessment takes into account:		
Age	80 (100.0)	Agreed in round 1
Gender	80 (100.0)	Agreed in round 1
Low BMI	78 (97.5)	Agreed in round 1
Family history of fragility fractures	77 (96.3)	Agreed in round 1
Treatment with glucocorticoids	79 (98.8)	Agreed in round 1
Smoking	76 (95.0)	Agreed in round 1
Alcohol consumption	75 (93.8)	Agreed in round 1
Secondary causes	77 (96.3)	Agreed in round 1
Loss of height ≥ 4 cm	78 (97.5)	Agreed in round 1
History of falls in the last 3 months	74 (92.5)	Agreed in round 1
15-20. To prevent new fragility fractures, the following are usually carried out:		
Health recommendations and changes in healthy lifestyle habits	80 (100.0)	Agreed in round 1
Active treatment (antiresorptive or bone-forming)	79 (98.8)	Agreed in round 1
Calcium supplementation	72 (90.0)	Agreed in round 1
Vitamin D supplementation	75 (93.8)	Agreed in round 1
Rehabilitation programmes	66 (82.5)	Agreed in round 1
Long-term patient follow-up programmes	71 (88.8)	Agreed in round 1
21-22. If bone densitometry is not available, it can be used:		
Validated fracture risk assessment tools	77 (96.3)	Agreed in round 1
X-ray of the lumbar and thoracic spine	72 (90.0)	Agreed in round 1
23-24. In the assessment of the risk of fracture:		
The FRAX® tool validated in the Spanish population is mainly used	73 (91.3)	Agreed in round 1
The use of new technologies is useful	73 (91.3)	Agreed in round 1

Abbreviations: FRAX = Fracture risk assessment tool

(1) In round 1, when panellists were asked to rate their level of agreement regarding the use of bone remodelling markers in the clinical evaluation for early diagnosis, the item failed to reach consensus given that these markers lack solid scientific evidence for their use, they are only used in selected cases, and it is complicated to use them in routine clinical practice. Therefore, the members of the scientific committee decided to reformulate this item into 3 new statements (items 11-13).

Supplementary Table 3: Results of the two-step Delphi process for the items regarding to the choice of the most appropriate treatment for the specific patient profile

Items	Accepted (scores 7-9) n (%)	Result of Delphi process
**Availability of current therapeutic alternatives**		
1. The patient receives information on the non-pharmacological and pharmacological therapeutic alternatives currently available according to the diagnosis of their osteoporosis.	74 (92.5)	Agreed in round 1
2. The patient receives information on the non-pharmacological therapeutic alternatives available, such as exercise and nutrition (calcium intake in the diet), among others.	76 (95.0)	Agreed in round 1
3-4.The choice between the different therapeutic alternatives available for osteoporosis is determined by:		
The criteria of each specialist who manages patients with this profile.	73 (91.3)	Agreed in round 1
Economic criteria in addition to therapeutic benefit.	60 (75.0)	Agreed in round 1
5. New commercially available treatments for osteoporosis are readily incorporated into the portfolio of available therapies.	72 (90.0)	Agreed in round 1
**Treatment lines / Optimization**		
1. In the different levels of categorisation of fracture risk, the first step of treatment is determined by the avoidance of possible risk factors (smoking and alcohol), the correction of possible nutritional deficiencies, mainly of calcium and vitamin D (including sun exposure), through dietary recommendations, pharmacological supplements (if necessary), and recommendations for exercise habits appropriate for each patient.	79 (98.8)	Agreed in round 1
2. In high and very high risk categorisations, it is necessary to determine the risk of falling and carry out the relevant actions for its prevention.	78 (97.5)	Agreed in round 1
3. In the case of the category of high risk of fracture, treatment with oral bisphosphonates or other antiresorptives should be considered as the first pharmacological option	69 (86.3)	Agreed in round 1
4. In the case of the category of very high risk of fracture, treatment with bone-forming drugs should be considered, and a sequential change to antiresorptive drugs should be assessed after one or two years	78 (97.5)	Agreed in round 1
5. In the very high fracture risk category, the simultaneous combination of a bone-forming drug with an antiresorptive drug may be considered	39 (50.0)	Not agreed in round 2 after being reformulated[1]

6. The monitoring criteria for the different therapeutic alternatives available are currently well defined	60 (75.0)	Agreed in round 1
7-10. The monitoring criteria for the different therapeutic alternatives available are defined by:		
Improvement in bone densitometry	70 (87.5)	Agreed in round 1
Improvement in bone remodelling markers	63 (78.8)	Agreed in round 1
Absence of new fractures	75 (93.8)	Agreed in round 1
Decrease in the risk of the appearance of new fractures	76 (95.0)	Agreed in round 1
11-13. Duration of treatment is currently well defined for:		
Oral and intravenous bisphosphonates	39 (50.0)	Not agreed in round 2 after being reformulated[(2)]
Teriparatide and biosimilars	76 (97.4)	Agreed in round 2 after being reformulated[(2)]
Denosumab	39 (50.0)	Not agreed in round 2 after being reformulated[(2)]
14-16. The duration of treatment with oral bisphosphonates is currently well defined at:		
5 years	56 (71.8)	Agreed in round 2 after being reformulated[(2)]
10 years	29 (37.2)	Not agreed in round 2 after being reformulated[(2)]
< 10 years	9 (11.5)	Not agreed in round 2 after being reformulated[(2)]
17-19. The duration of treatment with intravenous bisphosphonates is currently well defined at:		
3 years	54 (69.2)	Agreed in round 2 after being reformulated[(2)]
6 years	32 (41.0)	Not agreed in round 2 after being reformulated[(2)]
> 6 years	6 (7.7)	Not agreed in round 2 after being reformulated[(2)]
20-23. The duration of treatment with teriparatide is currently well defined at:		
< 1 year	7 (9.0)	Not agreed in round 2 after being reformulated[(2)]
1 year	10 (12.8)	Not agreed in round 2 after being reformulated[(2)]
2 years	74 (94.9)	Agreed in round 2 after being reformulated[(2)]
> 2 years	10 (12.8)	Not agreed in round 2 after being reformulated[(2)]
24-26. The duration of treatment with denosumab is currently well defined at:		
5 years	36 (46.2)	Not agreed in round 2 after being reformulated[(2)]
10 years	56 (71.8)	Agreed in round 2 after being reformulated[(2)]
> 10 years	18 (23.1)	Not agreed in round 2 after being reformulated[(2)]
27. The maximum duration defined for each treatment is met in patients in whom the	70 (87.5)	Agreed in round 1

therapeutic objective is achieved without adverse events		
28. Clinical practice guidelines agree in recommending sequential treatment starting with an bone-forming drug and continuing with a parenteral antiresorptive drug in patients at high risk of fracture	62 (79.5)	Agreed in round 2 after being reformulated[(3)]
**Biosimilar availability**		
1. Approved biosimilar treatments can be used in appropriate patients in the same way as bio-original treatments	79 (98.8)	Agreed in round 1
2. The choice of the corresponding biosimilar is safe and effective	79 (98.8)	Agreed in round 1
3. The choice of the corresponding biosimilar ensures that the therapeutic target is achieved in the same way as the bio-original treatment	77 (96.3)	Agreed in round 1
4. It is necessary to inform the patient that he/she will be treated with a biosimilar therapeutic alternative	31 (39.7)	Not agreed in round 2 after being reformulated[(4)]
**Adverse events**		
1. The common adverse events of the different therapeutic alternatives are clearly specified in clinical guidelines	79 (98.8)	Agreed in round 1
2. The adverse events of the different treatment alternatives determine the decision to choose one drug or another	75 (93.8)	Agreed in round 1

[(1)] In round 1, when panellists were asked to rate their level of agreement regarding the use of the treatment with antiresortives in combination with bone-forming therapies. To clarify any possible doubts, the members of the scientific committee decided to reformulate this item into a new statement clarifying that it is the simultaneous use of both treatments at the same time (item 5 of Treatment lines / Optimization).

[(2)] In round 1, when panellists were asked to rate their level of agreement regarding the current definition in terms of the duration of the different treatments available, the item reached consensus. However, the members of the scientific committee decided to reformulate and break down this item into several new statements in order to gather more detailed information (items 11-26 of Treatment lines / Optimization).

[(3)] In round 1, panellists were asked to rate their level of agreement regarding the order in the sequence of treatments for this patient profile, but in a very generic way. Consequently, the members of the scientific committee decided to reformulate this item into a new statement in order to gather more detailed information (item 28 of Treatment lines / Optimization).

[(4)] In round 1, panellists were asked to rate their level of agreement on patient notification when a biosimilar treatment is administered, and the item didn't reach consensus. Consequently, the members of the scientific committee decided to reformulate this item into a new statement in order to gather more detailed information on the need for such notification (item 4 of Biosimilar availability).

Supplementary Table 4: Results of the two-step Delphi process for the items regarding to the treatment-associated quality of life in patients with osteoporosis at high risk of fracture

Items	Accepted (scores 7-9) n (%)	Result of Delphi process
**Adherence to treatment**		
1. The decision regarding to the most appropriate therapeutic alternative at each moment should be agreed with the patient according to their preferences	76 (95.0)	Agreed in round 1
2. Patient adherence to treatment should be monitored appropriately in the case of high and very high risk osteoporosis	80 (100.0)	Agreed in round 1
3. Patient adherence to treatment for osteoporosis determines the choice of the most appropriate therapeutic alternative in each case	80 (100.0)	Agreed in round 1
4. Corrective action plans should be available for non-adherent patients to increase/improve adherence	78 (97.5)	Agreed in round 1
5-10. Patient adherence or non-adherence to osteoporosis treatment is determined by:		
Adverse events of each specific treatment	79 (98.8)	Agreed in round 1
Complexity of the treatment regimen	77 (96.3)	Agreed in round 1
Periodicity of treatment dosage	75 (93.8)	Agreed in round 1
Route of administration	76 (95.0)	Agreed in round 1
Duration of treatment	73 (91.3)	Agreed in round 1
Patient's polymedication	79 (98.8)	Agreed in round 1
11-12. The patient is more adherent:		
To oral treatments for osteoporosis	37 (46.3)	Not agreed in round 1 Eliminated(1)
To subcutaneous treatments for osteoporosis	66 (82.5)	Agreed in round 1
To intravenous treatments for osteoporosis	68 (85.0)	Agreed in round 1
**Patient care programmes**		
1. There is an established plan for the long-term management of the high and very high-risk osteoporosis patient	67 (83.8)	Agreed in round 1
2. A multidisciplinary team is involved in the long-term management of the patient with high and very high risk osteoporosis	58 (72.5)	Agreed in round 1
3-15. Taking into account the actual current experience, the team of a monographic osteoporosis practice / osteoporosis unit consists of:		
Traumatologist	44 (56.4)	Not agreed in round 2 after being reformulated(2)
Rheumatologist	72 (92.3)	Agreed in round 2 after being reformulated(2)
Internal medicine specialist	34 (43.6)	Not agreed in round 2 after being reformulated(2)

Specialist in geriatrics	44 (56.4)	Not agreed in round 2 after being reformulated(2)
Specialist nursing	56 (71.8)	Agreed in round 2 after being reformulated(2)
Primary care physician	51 (65.4)	Not agreed in round 2 after being reformulated(2)
Physiotherapist	28 (35.9)	Not agreed in round 2 after being reformulated(2)
Rehabilitator	41 (52.6)	Not agreed in round 2 after being reformulated(2)
Psychologist	11 (14.1)	Not agreed in round 2 after being reformulated(2)
Dentist	14 (17.9)	Not agreed in round 2 after being reformulated(2)
Nutritionist	25 (32.1)	Not agreed in round 2 after being reformulated(2)
Specific patient support staff	35 (44.9)	Not agreed in round 2 after being reformulated(2)
Representative of the osteoporosis patients' association	19 (24.4)	Not agreed in round 2 after being reformulated(2)
16-28. Regardless of what happens currently, the ideal team of an osteoporosis unit should consist of:		
Traumatologist	55 (70.5)	Agreed in round 2 after being reformulated(2)
Rheumatologist	73 (93.6)	Agreed in round 2 after being reformulated(2)
Internal medicine specialist	40 (51.3)	Not agreed in round 2 after being reformulated(2)
Specialist in geriatrics	65 (83.3)	Agreed in round 2 after being reformulated(2)
Specialist nursing	70 (89.7)	Agreed in round 2 after being reformulated(2)
Primary care physician	64 (82.1)	Agreed in round 2 after being reformulated(2)
Physiotherapist	48 (61.5)	Not agreed in round 2 after being reformulated(2)
Rehabilitator	59 (75.6)	Agreed in round 2 after being reformulated(2)
Psychologist	29 (37.2)	Not agreed in round 2 after being reformulated(2)
Dentist	28 (35.9)	Not agreed in round 2 after being reformulated(2)
Nutritionist	49 (62.8)	Not agreed in round 2 after being reformulated(2)
Specific patient support staff	53 (67.9)	Agreed in round 2 after being reformulated(2)
Representative of the osteoporosis patients' association	33 (42.3)	Not agreed in round 2 after being reformulated(2)
29-41. Taking into account the current actual experience, the team of a FLS consists of:		
Traumatologist	67 (85.9)	Agreed in round 2 after being reformulated(2)
Rheumatologist	69 (88.5)	Agreed in round 2 after being reformulated(2)

Internal medicine specialist	38 (48.7)	Not agreed in round 2 after being reformulated(2)
Specialist in geriatrics	52 (66.7)	Agreed in round 2 after being reformulated(2)
Specialist nursing	59 (75.6)	Agreed in round 2 after being reformulated(2)
Primary care physician	49 (62.8)	Not agreed in round 2 after being reformulated(2)
Physiotherapist	33 (42.3)	Not agreed in round 2 after being reformulated(2)
Rehabilitator	48 (61.5)	Not agreed in round 2 after being reformulated(2)
Psychologist	18 (23.1)	Not agreed in round 2 after being reformulated(2)
Dentist	15 (19.2)	Not agreed in round 2 after being reformulated(2)
Nutritionist	27 (34.6)	Not agreed in round 2 after being reformulated(2)
Specific patient support staff	36 (46.2)	Not agreed in round 2 after being reformulated(2)
Representative of the osteoporosis patients' association	21 (26.9)	Not agreed in round 2 after being reformulated(2)
42-54 . Regardless of what happens currently, the ideal team of a FLS should consist of:		
Traumatologist	71 (91.0)	Agreed in round 2 after being reformulated(2)
Rheumatologist	76 (97.4)	Agreed in round 2 after being reformulated(2)
Internal medicine specialist	46 (59.0)	Not agreed in round 2 after being reformulated(2)
Specialist in geriatrics	71 (91.0)	Agreed in round 2 after being reformulated(2)
Specialist nursing	74 (94.9)	Agreed in round 2 after being reformulated(2)
Primary care physician	71 (91.0)	Agreed in round 2 after being reformulated(2)
Physiotherapist	59 (75.6)	Agreed in round 2 after being reformulated(2)
Rehabilitator	65 (83.3)	Agreed in round 2 after being reformulated(2)
Psychologist	41 (52.6)	Not agreed in round 2 after being reformulated(2)
Dentist	33 (42.3)	Not agreed in round 2 after being reformulated(2)
Nutritionist	56 (71.8)	Agreed in round 2 after being reformulated(2)
Specific patient support staff	58 (74.4)	Agreed in round 2 after being reformulated(2)
Representative of the osteoporosis patients' association	40 (51.3)	Not agreed in round 2 after being reformulated(2)
55. Taking into account the current experience, a FLS monitors the management of the patient at high and very high risk of fragility fracture	56 (71.8)	Agreed in round 2 after being reformulated(2)
56. Regardless of what happens currently, a FLS should monitor the management of the patient at high and very high risk of fragility fracture	76 (97.4)	Agreed in round 2 after being reformulated(2)

57-59. Based on the current experience, FLSs monitor the management of the patient at high and very high risk of fragility fracture at the level of:		
Hospital	70 (89.7)	Agreed in round 2 after being reformulated(2)
Primary care centers	30 (38.5)	Not agreed in round 2 after being reformulated(2)
Intermediate care centres (if available)	28 (47.5)	Not agreed in round 2 after being reformulated(2)
60-62. Regardless of what happens currently, FLSs should monitor the management of the patient at high and very high risk of fragility fracture at the level of:		
Hospital	76 (97.4)	Agreed in round 2 after being reformulated(2)
Primary care centers	64 (82.1)	Agreed in round 2 after being reformulated(2)
Intermediate care centres (if available)	55 (82.1)	Agreed in round 2 after being reformulated(2)
63-65. Taking into account the current experience, the FLSs are coordinated by:		
Physician and nursing staff specialised in the management of patients with osteoporosis	71 (92.3)	Agreed in round 2 after being reformulated(2)
Primary care physician and nurses	23 (29.5)	Not agreed in round 2 after being reformulated(2)
Intermediate care staff (physicians and nurses)	20 (35.7)(3)	Not agreed in round 2 after being reformulated(2)
66-68. Regardless of what happens currently, FLSs should be coordinated by:		
Physician and nursing staff specialised in the management of patients with osteoporosis	77 (98.7)	Agreed in round 2 after being reformulated(2)
Primary care physician and nurses	53 (67.9)	Agreed in round 2 after being reformulated(2)
Intermediate care staff (physicians and nurses)	45 (70.3)(4)	Agreed in round 2 after being reformulated(2)
69. Based on the current experience, primary care centres have a specialised plan for the management of patients with high-risk osteoporosis.	14 (17.9)	Not agreed in round 2 after being reformulated(2)
70. Regardless of what happens currently, primary care centres should have a specialised plan for the management of patients with high-risk osteoporosis.	70 (89.7)	Agreed in round 2 after being reformulated(2)
71. Based on the current experience, the primary care physician for each patient is informed of the specific care plan for the management of high-risk osteoporosis.	37 (47.4)	Not agreed in round 2 after being reformulated(2)
72. Regardless of what happens currently, the primary care physician for each patient should be	75 (96.2)	Agreed in round 2 after being reformulated(2)

informed of the specific care plan for the management of high-risk osteoporosis		
73-79. Patient support programmes in the treatment of high and very high risk osteoporosis should include:		
Printed support and information material	77 (96.3)	Agreed in round 1
Audio-visual support and information material	70 (87.5)	Agreed in round 1
Telephone follow-up of the patient	71 (88.8)	Agreed in round 1
Possibility of direct telephone contact by the patient	73 (91.3)	Agreed in round 1
Information through internet websites	62 (81.3)	Agreed in round 1
A mobile phone application with information, alerts and contact details	67 (83.8)	Agreed in round 1
Possibility of face-to-face care in the patient's home by nursing staff	75 (93.8)	Agreed in round 1
80. Sufficient consultation time is available to teach patients how to use the devices to administer their osteoporosis treatments	29 (36.3)	Not agreed in round 1 Eliminated(1)
81-84. Training of the patient to handle treatment delivery devices should be carried out by:		
Specialist physician	41 (51.2)	Not agreed in round 1 Eliminated(1)
Nurses	79 (98.8)	Agreed in round 1
Specialised staff of a patient support programme	77 (96.3)	Agreed in round 1
Pharmacy Service	52 (65.0)	Not agreed in round 1 Eliminated(1)

Abbreviations: FLS = Fracture-liaison service.

(1) The removal of this item was due to the opinion of the scientific committee that the level of agreement was not going to change substantially in the round 2.

(2) In round 1, panellists were asked to rate their level of agreement regarding the structure of specialised units and FLS. However, the members of the scientific committee decided to reformulate and break down round 1 items into several new statements in order to gather more detailed information (items 3-72 of Patient care programmes).

(3) This item was evaluated by n= 56 due to the fact that intermediate care centers are only available in some regions in Spain.

(4) This item was evaluated by n= 64 due to the fact that intermediate care centers are only available in some regions in Spain.
